# How negative self-views may interfere with building positive relationships: An experimental analogue of identity dysfunction in borderline personality disorder

**DOI:** 10.1371/journal.pone.0301196

**Published:** 2024-03-28

**Authors:** Charlotte C. van Schie, Laura Whiting, Brin F. S. Grenyer

**Affiliations:** School of Psychology, University of Wollongong, Wollongong, New South Wales, Australia; University of Glasgow, UNITED KINGDOM

## Abstract

**Introduction:**

A disturbed, negative sense of self is associated with various interpersonal difficulties and is characteristic of disorders such as borderline personality disorder (BPD). Negative self-views may affect an individuals’ ability to build positive relationships, including a therapeutic relationship. However, it is not yet well understood how identity disturbances give rise to interpersonal difficulties. Using an experimental analogue design, we tested whether identity disturbances are associated with interpersonal difficulties.

**Methods:**

Participants were university students (*N* = 43, age *M* = 20.51 (*SD* = 3.08), women *N* = 32 (74.4%)) who reported moderate to high levels of BPD features, with 34.9% reporting significant BPD features as measured by the Borderline scale of the Personality Assessment Inventory (PAI-BOR). In a within-subject experimental paradigm using a Social Feedback Task, participants received negative, intermediate, and positive evaluations, supposedly from a panel. Using multilevel models, we tested whether negative self-views were associated with how much the participants liked, trusted, and felt close to each of the three panel members who provided either predominantly negative, intermediate, or positive feedback.

**Results:**

People with more negative self-views reported lower mood in response to positive feedback. In addition, where people with more positive self-views felt better when receiving feedback that was congruent with their self-views, people with more negative self-views did not report a better mood. Importantly, people with negative self-views felt lower desire to affiliate with the member who provided predominantly positive feedback. Affiliation was not affected when feedback was given by the negative member and intermediate member to those with negative self-views.

**Conclusions:**

The findings validated that those with more negative self-views anticipated and expected more negative responses from others. Negative self-views, as relevant for BPD, may explain how people relate differently to those giving different types of feedback. Pervasive negative self-views may interfere with building new relationships including the therapeutic alliance. It may be helpful for clinicians to be aware of the potential challenges around creating a supportive therapeutic relationship for patients with negative self-views. Overly positive affirmations made by clinicians may inadvertently lower the patient’s mood and may impede alliance formation.

## Introduction

A coherent self allows individuals to experience a sense of continuity over time and across different social contexts, and has shown to be important for psychological wellbeing and the formation of healthy interpersonal relationships [1, 2]. In contrast, a disturbed, negative sense of self has found to be characteristic of a wide range of psychological disorders and has shown to be associated with various interpersonal difficulties [3]. However, it is not yet well understood how identity disturbances give rise to interpersonal difficulties. Therefore, this study aims to investigate how identity disturbances, specifically negative self-views, relate to difficulties in affiliating with others.

Theoretical research argues that the way an individual views themselves consequently shapes how they relate to others [4–6]. It is thought that as children develop, they internalise representations of self and other through their early experiences with attachment figures [7]. Early adverse social experiences, such as childhood maltreatment, are thought to contribute to the development of an unstable, fragile and negative sense of self [8, 9]. These negative models of self often become pervasive patterns of relating to the self and to others [10] and can impair learning new information about the self particularly when this is different from the existing self-view [11]. Individuals prefer feedback that confirms their self-views, irrespective of whether it is positive or negative, because this confirmation enables them to maintain a reliable understanding of themselves that fosters a sense of self-continuity [12]. Thus, the process of self-verification may be important in explaining how self-views guide which social feedback should be integrated and which should be disregarded as irrelevant [13].

Research has shown that those who hold predominantly negative self-views actively seek out negative feedback from others and often prefer friends and romantic partners who evaluate them negatively [14]. Moreover, a study showed that when people with negative self-views make positive self-statements, it can adversely affect their mood and self-esteem [15]. Additionally, studies have found that when individuals are not being confirmed in their self-view, they can feel less trusting towards others and may be more likely to divorce from their partners [16, 17]. Therefore, this self-verification process may not only reinforce a negative sense of self, but may also interfere with relating to others [16, 18].

Identity disturbances and interpersonal difficulties are the core criteria for personality disorder [19]. In particular, individuals with Borderline Personality Disorder (BPD) often hold pervasive and negative self-views, commonly seeing themselves as inherently bad, evil or boring people [20–22]. Moreover, individuals with BPD have difficulty in forming and maintaining positive and enduring interpersonal relationships [23–25]. These self and interpersonal difficulties have been expressed as significant impairments in processing social information, in particular, social feedback about the self [26]. Individuals with BPD have a tendency to disproportionately attribute blame for negative occurrences to themselves [27–29] and struggle to effectively engage with positive social feedback such as being socially accepted [30]. A study has even shown that people high in BPD features prefer to receive unfavourable as opposed to favourable self-relevant feedback from others [31]. Moreover, individuals with BPD experience low affiliation with others in situations of social inclusion [32, 33] and have difficulty developing trust in others [30, 34–36], including the therapeutic relationship [37–39]. However, although various studies have shown that altered social feedback processing occurs in BPD, it is unclear whether negative self-views contribute to difficulties in engaging with positive feedback from others and forming positive relationships. Furthering our understanding of how negative self-views may contribute to interpersonal difficulties is important as many individuals with BPD experience remaining challenges in identity functioning and maintaining meaningful relationships, despite the effectiveness of evidence-based interventions [40–46].

In sum, it is proposed that individuals with negative self-views, such as those with BPD, may have more difficulty with processing positive (incongruent) social feedback, which may make it harder for them to relate to the person providing the feedback. It remains unclear how individuals with negative self-views may affiliate with others that provide negative feedback, which is potentially self-verifying. Based on previous research, we expect one of two outcomes. Firstly, individuals with higher negative self-views will affiliate more with the panel member providing predominately negative feedback as this member confirms their self-views. Alternatively, negative self-views will not predict affiliation with the predominantly negative member given the negative effects this feedback may have on individuals.

To date, there has been limited empirical research examining the role that negative self-views play in interpersonal difficulties as relevant to BPD. The findings could have implications for individuals’ relationships with their friends, families, and romantic partners but also for the therapeutic relationship. Considering that the establishment of trust and mutual relation between the patient and clinician is essential for promoting therapeutic change [10, 47], understanding how negative self-views interfere with affiliating with others is paramount.

Therefore, the aim of the current study is to investigate how negative self-views influence the way in which individuals affiliate with others when provided with social feedback. We aim to do this through using a panel version of the previously validated Social Feedback Task [48] which includes three panel members who provide either mainly positive, mainly negative, or mainly intermediate character trait feedback. The study will focus on assessing individuals with varying levels of self-reported BPD features. For identity development, adolescence as well as early adulthood are important life phases [49, 50]. Moreover, the onset and development of BPD is commonly reported to be in adolescence and early adulthood [51, 52]. We therefore aimed to recruit participants in these life phases. However, we used convenience sampling and the age range was therefore restricted to be between 18 and 30 years.

We identified three constructs to measure affiliation that are relevant in the context of this task, i.e., closeness, trust and liking. Our main aim is to measure a sense of closeness that participants may experience in relation to the panel members. Based in attachment theory, the proximity to others, i.e. feeling close, is relevant in building interpersonal relations [53]. The feeling of closeness has been shown to be strongly associated with affiliation with others [54, 55] and is therefore included as an important measure. In terms of clinical application, trust is essential in establishing the therapeutic alliance [10, 47]. Moreover, previous studies have shown that trust is impaired in BPD, and is affected by self-verification processes [16, 30]. Lastly, we included liking as a construct often used in various tasks where social feedback is received or given and has shown to be easily endorsable in settings where participants interact with new people (e.g. [56, 57]).

In all, this study aims to test the following hypotheses:

1. Negative self-views will be positively related to self-reported BPD features in the current sample.
2. In the panel version of the Social Feedback Task, people will report a better mood after positive feedback and worse mood after negative feedback compared to intermediate feedback. Moreover, people will report a better mood for more congruent feedback, particularly for congruent negative feedback.
3. People with more negative self-views will have lower mood in response to positive feedback compared to intermediate feedback.
4. People with more negative self-views will report less affiliation, i.e., closeness, trust and liking, with the panel member providing predominately positive feedback compared to the intermediate member.
5. People with more negative self-views will report either no altered affiliation or more affiliation with the panel member providing predominantly negative feedback compared to the intermediate member.

## Methods

### Participants

Participants (*N* = 43, age *M* = 20.51, *SD* = 3.08, *range* = 18–29) were university students who were recruited via notices put up around the University of Wollongong (UOW) targeting those studying undergraduate psychology subjects. The sample comprised of 32 women (74.4%), 11 men (25.6%), see Table 1. Individuals had to be between 18 and 30 years of age and proficient in English to participate. Use of certain psychotropic medications were excluded, i.e., benzodiazepines (equivalent of > 20 mg of oxazepam) and antipsychotics. Mood responses to the Social Feedback Task were checked for flat responding (i.e., variance in mood responses equals 0) and three participants were excluded, resulting in the sample size of *N* = 43 as described above. Participants were reimbursed for their participation with either course credits or being eligible to receive one of two $50 vouchers. All participants gave both verbal and electronic informed consent to participate. The study was granted approval by the Human Research Ethics Committee at UOW (2019/449).

**Table 1:** Participant demographics and self-reported psychiatric diagnoses (N = 43).

Baseline characteristics	*N* (%)
**Age** (years) *M* (*SD*)	20.51 (3.08) (*range*: 18–29)
**Sex**	
Women	32 (74.4%)
Men	11 (25.6%)
**Highest Education Level**	
High School Completion	34 (79.1%)
Vocational Training	5 (11.6%)
Higher Education (University Degree)	4 (9.3%)
**Relationship Status**	
Single	24 (55.8%)
In a relationship, unspecified	15 (34.9%)
Married	1 (2.3%)
In a de-facto relationship	3 (7.0%)
**Occupation**	
Work or study full-time	35 (81.4%)
Work or study part-time	8 (18.6%)
Not currently working or studying	0 (0%)
**Psychiatric Disorder–nr of diagnoses**	[any *N* = 18 (41.9%)]
1 diagnosis	5 (11.6%)
2 diagnoses	9 (20.9%)
3 or more diagnoses	4 (9.3%)
**Psychiatric Disorder—past or current diagnosis**	
Mood disorder	13 (30.2%)
Anxiety disorder	14 (32.5%)
Post-traumatic stress disorder	1 (2.3%)
Attention deficit/hyperactivity disorder	3 (7.0%)
Autism spectrum disorder	1 (2.3%)
Eating or Body dysmorphic disorder	2 (4.6%)
Borderline personality disorder	1 (2.3%)

*Note*. *M* = mean, *SD* = standard deviation, *N* = number of individuals with characteristic, % = percentage of individuals with characteristic.

The lifetime prevalence of a self-reported psychiatric disorder in the sample was 41.9%, see Table 1. Moderate to high levels of self-reported BPD features were present in the sample (*M* = 31.49, *SD* = 10.96), as measured by the PAI-BOR (see measures and materials for description of all instruments), see Table 2. The prevalence of clinically significant BPD features was 34.9% (*N* = 15), as indicated by a score higher than 37 on the PAI-BOR [58]. There were relatively high levels of identity incoherence in the sample (*M* = 81.98, *SD* = 26.88), as indicated by the SCIM scores (see measures and materials), and in comparison to previous studies using similar non-clinical samples ([59]; M = 68.71, SD = 21.18). This sample may have reported slightly elevated levels of psychopathology due to the time of data collection being during the Covid-19 pandemic (June 2021 –August 2021).

**Table 2:** Means, standard deviations, and correlations with confidence intervals of measures of BPD features and identity disturbances (N = 43).

Variable	*M*	*SD*	1	2	3	4	5	6
1. BPD Features (total PAI-BOR)	31.49	10.96						
2. Negative self-views	3.27	0.90	.58**					
			[.33, .75]					
3. Positive self-views	5.76	0.74	-.24	-.41**				
			[-.51, .06]	[-.63, -.13]				
4. Identity incoherence (total SCIM)	81.98	26.88	.61**	.57**	-.30			
			[.38, .77]	[.33, .74]	[-.55, .00]			
5. Consolidated identity (subscale SCIM, reversed)	31.37	8.99	.60**	.62**	-.55**	.81**		
			[.37, .76]	[.39, .78]	[-.73, -.30]	[.67, .89]		
6. Disturbed identity (subscale SCIM)	33.98	13.48	.38*	.35*	-.09	.86**	.46**	
			[.09, .61]	[.05, .58]	[-.38, .22]	[.76, .92]	[.18, .67]	
7. Lack of identity (subscale SCIM)	16.63	8.93	.66**	.57**	-.21	.89**	.74**	.63**
			[.45, .80]	[.33, .74]	[-.48, .10]	[.81, .94]	[.56, .85]	[.41, .78]

*Note*. *M* and *SD* are used to represent mean and standard deviation, respectively. Values in square brackets indicate the 95% confidence interval.

* indicates p < .05.

** indicates p < .01.

### Procedure

**Fig 1:**
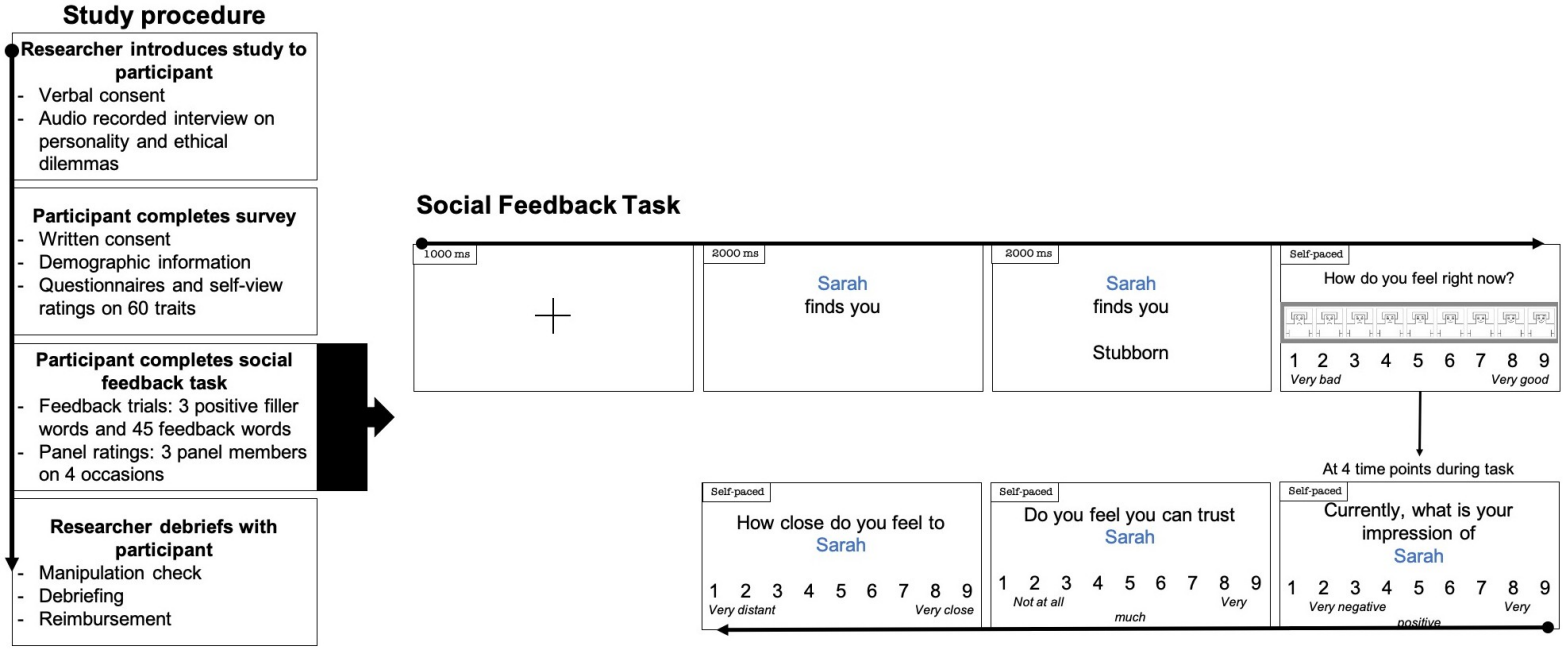
Study procedure and displays and timings of a trial in the panel Social Feedback Task. Participants were asked to rate their level of affiliation to the panel members after trial 9, 21, 33 and 45.

At recruitment, participants were informed about the study, provided verbal informed consent to participate and were interviewed as part of the Social Feedback Task, see Fig 1. Participants then completed an online survey where they provided written informed consent, demographic information, medical history including psychiatric diagnoses, and answered several questionnaires (see measures and materials). Next, participants completed the Social Feedback Task. In closing, a manipulation check was conducted to determine whether participants believed the Social Feedback Task cover story (see Supplementary information). Participants were also debriefed about the set-up of the study including the predetermined feedback and had the opportunity to discuss their study experiences before being reimbursed.

### Measures and materials

#### Personality Assessment Inventory- Borderline Scale (PAI-BOR)

The PAI-BOR is a self-report scale, which was used to measure features of BPD [58]. The 24-item instrument assesses four core symptoms of BPD pathology: affective instability, identity disturbances, negative interpersonal relationships, and self-harm/impulsivity. Each of the four subscales consist of 6 items and participants are asked to rate each item on a 4-point Likert scale from *0 (False)* to *3 (Very true)*. The total score, calculated by summating the scores of each subscale, was used in the current study (selected items reverse-scored). Higher scores indicate higher BPD features, with a total raw score of 37 or higher suggesting the presence of significant BPD symptoms [58]. The PAI-BOR has found to be a valid and reliable measure of BPD features which has been widely used in university, community, and clinical samples [60–62]. Test-retest reliability in clinical and non-clinical samples has previously been estimated at 0.73 [63]. In the current study, the PAI-BOR demonstrated good reliability, *α* = .86.

#### Self-Concept and Identity Measure (SCIM)

The SCIM is a dimensional self-report measure which assesses both healthy identity functioning and clinically relevant identity disturbance [59]. The 27-item scale is divided into three subscales: consolidated identity, disturbed identity, and lack of identity. The consolidated identity subscale items encompass a sense of knowing who one is and the healthy experiences of feeling integrated and whole, and connected to one’s past. The disturbed identity subscale assesses identity incoherence and fragmentation, as the items focus on measuring uncertainty and confusion in knowing who one is and inconsistencies in self-beliefs and values. The lack of identity subscale measures feelings of emptiness and brokenness. Example items include “I know what I believe and value” (consolidated), “I change a lot depending on the situation” (disturbed) and “I feel lost when I think about who I am” (lacking). All items are scored on a 7-point Likert scale ranging from *1 (Strongly disagree)* to *7 (Strongly agree)*. The total score, which was used in the current study, is calculated by reverse scoring the consolidated identity subscale and summing this score with the disturbed and lack of identity subscale scores. Higher total scores are indicative of greater identity disturbance and incoherence. The SCIM is a relatively novel measure however has been found to have strong reliability (non-clinical sample α = 0.88 [59]) when assessing problematic identity functioning in both clinical and non-clinical samples [3, 64]. Currently, there is no normative data. In non-clinical samples *M* = 68.71, *SD* = 21.18 of total SCIM score has been found [59]. Identity problems on the SCIM have also shown to correlate highly with BPD [65]. In the current study, the SCIM demonstrated excellent reliability, *α* = .93.

#### Social Feedback Task—Panel version

The Social Feedback Task is an experimental paradigm adapted from van Schie, Chiu [48], which measures people’s responses to social feedback. In this task, participants were firstly required to engage in a brief interview where they answered a series of personal questions and responded to moral dilemmas. The interview questions were specially designed to elicit both positive and negative characteristics from participants (see Supplementary information). The interview was audio-recorded with the participant’s consent and participants were informed that their interview would be listened to by a panel of three expert members from the psychology research team. Participants were told that the panel members would provide their ‘impressions’ of them through evaluative feedback based off their interview answers. Specifically, participants were informed that the panel members would choose from a list of personality character traits. In reality, however, all participants received the same feedback.

In total, 45 feedback words were presented one by one in random order; 15 positive (e.g., kind), 15 intermediate (e.g., practical) and 15 negative (e.g., boring) words, see S1 Table in S1 File. The feedback words were validated in a previous study and the English translation of feedback words was pilot tested in terms of their valence (N = 18) [48]. Words were selected based on valence rating with intermediate feedback being relatively neutral and with positive and negative words being more strongly rated as respectively positive or negative. One panel member provided predominantly positive feedback, another member provided predominantly negative feedback, and the final member provided mostly intermediate feedback. The distribution of positive, intermediate, and negative feedback each member provided is shown in S2 Table in S1 File. Participants were, however, unaware of this distribution.

It was indicated to participants by name and colour which panel member was providing feedback. Names of panel members were counterbalanced so that each name was associated with the negative, intermediate, and positive member in the panel. With three names across three valances, there were six counterbalance orders, see S3 Table in S1 File. Participants were equally distributed over the six counterbalance orders (χ^2^ (5) = 3.186, *p* = .671). Panel member names were sex congruent with the participant to not introduce potential variation in responses due to receiving feedback from sex incongruent panel member. The feedback words were not repeated, and panel members did not appear twice in a row. The task started with three positive filler words so that all panel members began on a positive note. These filler words were not included in the analyses.

Fig 1 shows the display of a single trial. After each feedback word, participants were asked to rate how they felt at that moment (mood) from *1 (Very good)* to *9 (Very bad)*. Moreover, the 45 feedback trials were divided into four blocks (1–9, 10–21, 22–33, 34–45). After each block, i.e., at four time points throughout the task, participants rated their affiliation with each of the three panel members in terms of liking on a scale ranging from 1 (*Very negative*) to 9 (*Very positive*), how much they trusted each panel member on a scale ranging from *1 (Not at all)* to *9 (Very much)* and how close they felt to each panel member on a scale ranging from *1 (Very distant) to 9 (Very close)*.

The task was programmed in PsychoPy (v2021.2.3) [66]. The panel version of the Social Feedback Task differed from previous studies [20, 48] in that the feedback did not come from one confederate but was (supposedly) provided by three panel members. Brief piloting took place in order to prove the viability of the new task modifications. Specifically, pilot participants were able to distinguish the panel members well enough to answer questions surrounding their affiliation with each. Based on the manipulation check, 86% of participants believed the set-up of the study (*N* = 37). All participants were included in the analysis.

#### Self-views measure

Before the social feedback was presented and as part of the questionnaires, participants rated how applicable 60 traits were to them on an 8-point Likert scale ranging from *1 (Not at all applicable to me)* to *8 (Very applicable to me*). The measure consisted of 20 positive, 20 intermediate and 20 negative traits (inclusive of the 45 feedback words). Mean applicability ratings of positive and negative words were used to determine the amount of positive and negative self-views each participant endorsed. The measure showed good reliability for both negative self-views (*α* = .87) and positive self-views (*α* = .85).

### Data analysis

To validate the panel version of the Social Feedback Task, we used multilevel analysis to model how mood after each feedback word (outcome) is affected by the valence of the feedback word (first level; negative, intermediate, positive), the applicability of the feedback word to the self (first level; applicability rating 1 to 8) and the panel member providing the feedback (first level; negative, intermediate, positive), including participant number as random effect. We used chi-square tests to compare models with main and interaction effects. First, a model with main effects only was compared to the null model. Next, the previously observed two-way interaction of feedback valence by applicability was added. Finally, it was tested whether the kind of panel member had a significant interaction effect with feedback valence and with applicability. Any non-significant interaction effects were not included in further modelling. The intermediate feedback was set as the reference category to compare to the negative and positive feedback. The member providing intermediate feedback was set as the reference category to compare to the predominantly negative and positive member.


$$\begin{array}{ll} Mood_{ij} & =\;\gamma_{00}+\;\gamma_{10}{\left(Negative\;valence\right)}_{ij}+\;\gamma_{20}{\left(Positive\;valence\right)}_{ij}+\gamma_{30}{\left(Applicability\right)}_{ij}\\ & +\;\gamma_{40}{\left(Negative\;member\right)}_{ij}+\;\gamma_{50}{\left(Positive\;member\right)}_{ij}\\ & +\gamma_{60}{\left(Negative\;valence\right)}_{ij}{\left(Applicability\right)}_{ij}\\ & +\gamma_{70}{\left(Positive\;valence\right)}_{ij}{\left(Applicability\right)}_{ij}\\ & +\gamma_{80}{\left(Negative\;member\right)}_{ij}{\left(Applicability\right)}_{ij}\\ & +\gamma_{90}{\left(Positive\;member\right)}_{ij}{\left(Applicability\right)}_{ij}\\ & +\gamma_{100}{\left(Negative\;valence\right)}_{ij}{\left(Negative\;member\right)}_{ij}\\ & +\gamma_{110}{\left(Positive\;valence\right)}_{ij}{\left(Negative\;member\right)}_{ij}\\ & +\gamma_{120}{\left(Negative\;valence\right)}_{ij}{\left(Positive\;member\right)}_{ij}\\ & +\gamma_{130}{\left(Positive\;valence\right)}_{ij}{\left(Positive\;member\right)}_{ij}+\upsilon_{0j}+\varepsilon_{ij} \end{array}$$

To understand how negative self-views may affect participants’ mood responses to feedback, we added the main effect of negative self-views on the second level and tested the two-way cross-level interaction effects of negative self-views by feedback valence, by applicability and by panel member.


$$\begin{array}{ll} Mood_{ij} & =\;\gamma_{00}+\;\gamma_{10}{\left(Negative\;valence\right)}_{ij}+\;\gamma_{20}{\left(Positive\;valence\right)}_{ij}+\gamma_{30}{\left(Applicability\right)}_{ij}\\ & +\;\gamma_{40}{\left(Negative\;member\right)}_{ij}+\;\gamma_{50}{\left(Positive\;member\right)}_{ij}\\ & +\gamma_{01}{\left(Negative\;selfview\right)}_{j}+\gamma_{60}{\left(Negative\;valence\right)}_{ij}{\left(Applicability\right)}_{ij}\\ & +\gamma_{70}{\left(Positive\;valence\right)}_{ij}{\left(Applicability\right)}_{ij}\\ & +\gamma_{11}{\left(Negative\;selfview\right)}_{j}{\left(Negative\;valence\right)}_{ij}\\ & +\gamma_{21}{\left(Negative\;selfview\right)}_{j}{\left(Positive\;valence\right)}_{ij}\\ & +\gamma_{31}{\left(Negative\;selfview\right)}_{j}{\left(Applicability\right)}_{ij}\\ & +\gamma_{41}{\left(Negative\;selfview\right)}_{j}{\left(Negative\;member\right)}_{ij}\\ & +\gamma_{51}{\left(Negative\;selfview\right)}_{j}{\left(Positive\;member\right)}_{ij}+\upsilon_{0j}+\varepsilon_{ij} \end{array}$$

To understand how negative self-views may affect how participants relate to the three panel members, we used multilevel analysis with negative self-view (second level; mean applicability of negative words per participant), and member (first level; negative, intermediate, and positive member) as predictors on three affiliation outcomes assessed at four time points during the task: level of closeness, level of trust, and degree of liking. To test whether participants related differently to each of the panel members overall, a model with the main effect of panel member was compared to the null model. Next, we added the main effect of negative self-views which would indicate whether people with more negative self-views relate differently to the panel members overall. Finally, we tested the two-way interaction of negative self-view by member which would indicate whether people with more negative self-views relate differently to the negative, intermediate and/or the positive member. The intermediate member was set as the reference level to compare to the positive and negative member. As there were three affiliation outcomes, we used a Bonferroni correction on the chi-square tests for model comparisons and evaluated *p*-values at alpha = 0.017. Note that below model is equivalent for the outcomes trust and liking.


$$\begin{array}{ll} Closeness_{ij} & =\gamma_{00}+\;\gamma_{10}{\left(Negative\;member\right)}_{ij}+\;\gamma_{20}{\left(Positive\;member\right)}_{ij}\\ & +\gamma_{01}{\left(Negative\;selfview\right)}_{j}+\gamma_{11}{\left(Negative\;selfview\right)}_{j}{\left(Negative\;member\right)}_{ij}\\ & +\gamma_{21}{\left(Negative\;selfview\right)}_{j}{\left(Positive\;member\right)}_{ij}+\upsilon_{0j}+\varepsilon_{ij} \end{array}$$

Data were analysed in R (version 4.0.2) with R studio (version 1.3.1093) using the packages psych (version 2.1.6) and lme4 (version 1.1–27.1). Data were plotted using effects (4.2–0) [67–69]. Multi-level analysis was used with maximum likelihood estimation. Model comparisons with chi square tests were used to test significance of main and interaction effects. Significance of parameter estimates within a model were determined using 95% confidence intervals bootstrapped with 5000 simulations. For effect sizes, we report standardized effect parameters (*std*.*b*) indicating the amount of change in the outcome with one standard deviation change in the predictor. For the full model we report the variance explained in relation to the null model (*f*^*2*^) [70].

## Results

Overall, participants with more self-reported BPD features reported more negative self-views (*r* = 0.58) and greater identity incoherence (*r* = 0.61), see Table 2. Moreover, participants with more negative self-views also reported greater identity incoherence (*r* = 0.57).

### Validation of social feedback task—Panel version

Multilevel analysis of the mood ratings during the Social Feedback Task indicated that the main effects of feedback valence, applicability and panel member were a significant improvement compared to the null model (*χ*^*2*^ (5) = 1132.40, *p* < .001). Adding the interaction effect of feedback valence by applicability was significant (*χ*^*2*^ (2) = 11.84, *p* = .003). Adding the two-way interaction effects of panel member by feedback valence and panel member by applicability did not significantly improve the model (*χ*^*2*^ (6) = 6.99, *p* = .322). In other words, mood was affected by feedback valence, applicability, and a feedback valence by applicability interaction, in line with our hypotheses. In addition, this panel version of the feedback task showed that panel member affects mood irrespective of feedback valence and applicability of the feedback.

**Fig 2:**
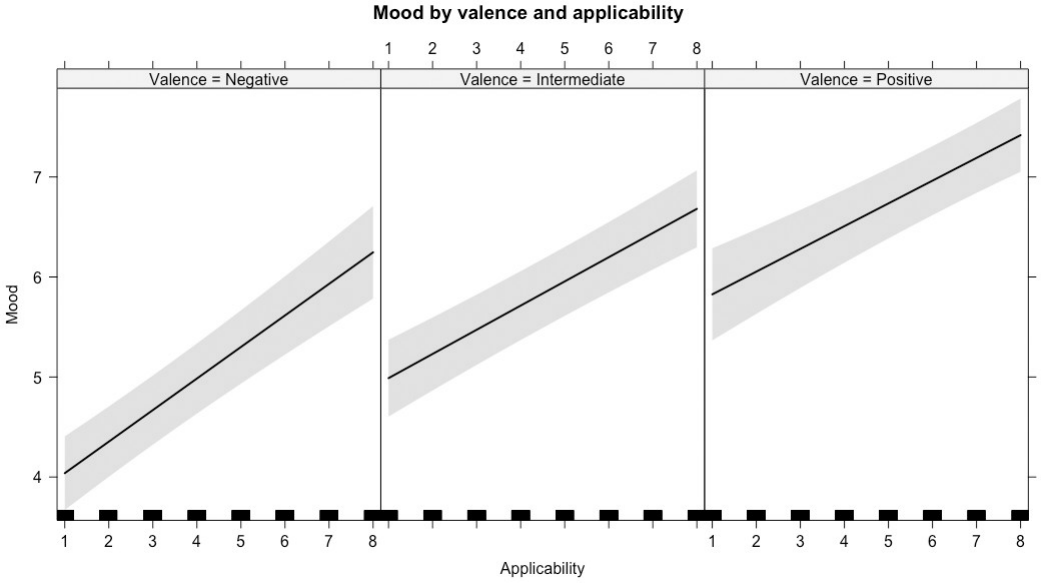
Participants’ mood when receiving feedback depends on the feedback valence (negative, intermediate, or positive) and how applicable the feedback is to the self (rating 1 to 8). Line depicts predicted mood responses. Margins around the line indicate 95% CI. Distribution of applicability ratings is plotted along x-axis.

Specifically, mood was lower for negative compared to intermediate feedback words (*b* = -1.19, *SE* = 0.16, *t* = -7.30, *95% CI* [-1.50, -0.88], *std*.*b* = -0.28) and higher for positive compared to intermediate feedback (*b* = 0.80, *SE* = 0.23, *t* = 3.43, *95% CI* [0.35, 1.25], *std*.*b* = 0.19). Feedback that was more applicable to the self was related to a better mood (*b* = 0.26, *SE* = 0.03, *t* = 10.25, *95% CI* [0.21, 0.31], *std*.*b* = 0.27). The two-way interaction of feedback valence by applicability indicated that particularly applicable negative feedback related to better mood (*b* = 0.12, *SE* = 0.04, *t* = 3.19, *95% CI* [0.05, 0.19], *std*.*b* = 0.11), see Fig 2. In addition, there was a main effect of panel member, indicating that receiving feedback from the negative member lowered mood regardless of the feedback valence this member provided (*b* = -0.16, *SE* = 0.08, *t* = -2.01, *95% CI* [-0.32, -0.01], *std*.*b* = -0.04). The variance explained in this model was *R*^*2*^ = 0.31, indicating a large effect size, *Cohen’s f*^*2*^ = 0.45. For all model parameters, see S4 Table in S1 File.

### Negative self-views affect mood responses to feedback

**Fig 3:**
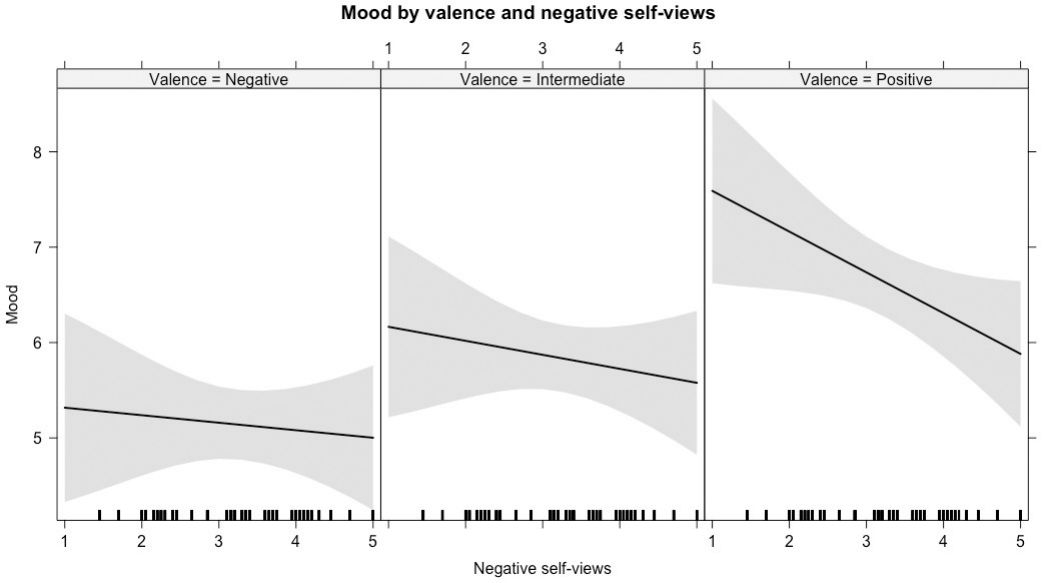
Negative self-views affect mood for negative, intermediate, and positive feedback. Line depicts predicted mood responses. Margins around the line indicate 95% CI. Distribution of negative self-views is plotted along x-axis.

Using multilevel models, we tested whether more negative self-views related to mood ratings in response to the feedback. Adding the main effect of negative self-views was not significant (*χ*^*2*^ (1) = 1.62, *p* = .203). However, the two-way interactions of negative self-view by feedback valence and of negative self-views by applicability was significant (*χ*^*2*^ (5) = 87.61, *p* < .001). In line with our hypotheses, this model indicated that people with more negative self-views have a lower mood after positive feedback (*b* = -0.28, *SE* = 0.09, *t* = -3.04, *95% CI* [-0.47, -0.10], *std*.*b* = -0.23), see Fig 3. Moreover, where in general, more applicable feedback related to a better mood, this was less strong for people with more negative self-views (*b* = -0.10, *SE* = 0.02, *t* = -5.45, *95% CI* [-0.14, -0.06], *std*.*b* = -0.42). In sum, more negative self-views related to lower mood after positive feedback and after feedback that was congruent with the self-view. For all model parameters, see S5 Table in S1 File. The additional variance explained by this model including negative self-views was *R*^*2*^ = 0.04, indicating a small effect size, *Cohen’s f*^*2*^ = 0.04.

### Negative self-views affect affiliation with the panel members

Using multilevel models, we tested whether the degree of negative self-views was associated with how much the participants affiliated with each of the three panel members.

**Fig 4:**
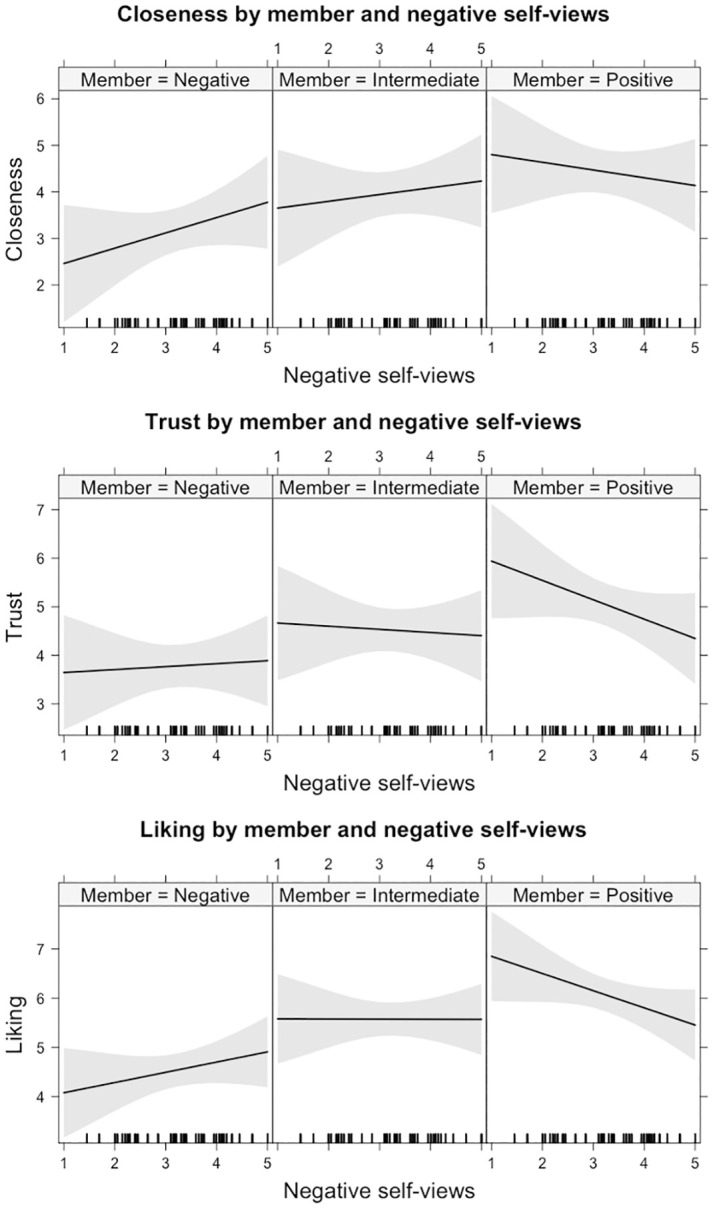
Level of affiliation with the positive, intermediate, and negative panel member in terms closeness, trust and liking, depends on negative self-views. Lines depict predicted affiliation ratings. Margin around the line indicates 95% CI. Distribution of negative self-views is plotted along x-axis.

There was a main effect of panel member on closeness ratings (*χ*^*2*^ (2) = 73.27, *p* < .001), no main effect of negative self-views on closeness (*χ*^2^ (1) = 0.17, *p* = .678), whereas the interaction between negative self-views and panel member was significant (*χ*^2^ (2) = 10.36, *p* = .006). Overall, participants felt less close to the negative member (*b* = -1.37, *SE* = 0.52, *t* = -2.63, *95% CI* [-2.41, -0.34], *std*.*b* = -0.33) and more close to the positive member (*b* = 1.46, SE = 0.52, *t* = 2.79, *95% CI* [0.42, 2.49], *std*.*b* = 0.35) compared to the intermediate member. The interaction effect indicated that as participants had more negative self-views, they felt less close to the positive member (*b* = -0.31, *SE* = 0.15, *t* = -2.01, *95% CI* [-0.61, -0.01], *std*.*b* = -0.26) compared to the intermediate member, see Fig 4. Negative self-views did not affect closeness to the negative member compared to the intermediate member (*b* = 0.18, *SE* = 0.15, *t* = 1.19, *95% CI* [-0.12, 0.49], *std*.*b* = 0.15). In other words, participants felt overall closer to the positive member. However, confirming our hypotheses, participants with more negative self-views felt less close to the positive member. The variance explained in the model predicting closeness by panel member, negative self-views and their interaction was *R*^*2*^ = 0.08, indicating a small effect size, *Cohen’s f*^*2*^ = 0.08. For all model parameters, see S6 Table in S1 File.

Regarding trust, there was a main effect of panel member (*χ*^*2*^ (2) = 65.42, *p* < .001), no main effect of negative self-views (*χ*^2^ (1) = 0.35, *p* = .553) and an interaction effect of negative self-views by member (*χ*^2^ (2) = 7.91, *p* = .019). Overall, participants trusted the positive member more (*b* = 1.61, *SE* = 0.57, *t* = 2.83, *95% CI* [0.44, 2.72,], *std*.*b* = 0.39) compared to the intermediate member. Trust in the negative member did not differ from the intermediate member (*b* = -1.14, *SE* = 0.57, *t* = -2.01, *95% CI* [-2.27, 0.01], *std*.*b* = -0.28). Although, the interaction effect did not withstand multiple correction, it is noteworthy that effects are in the hypothesised direction. That is, participants with more negative self-views tend to report lower trust in the positive member (*b* = -0.33, *SE* = 0.17, *t* = -1.99, *95% CI* [-0.66, 0.01], *std*.*b* = -0.28), see Fig 4. The variance explained in the model predicting trust by panel member, negative self-views and their interaction was *R*^*2*^ = 0.08, indicating a small effect size, *Cohen’s f*^*2*^ = 0.09.

Finally, with respect to degree of liking, there was a main effect of panel member (*χ*^2^ (2) = 94.62, *p* < .001), no main effect of negative self-views (*χ*^2^ (1) = 0.09, *p* = .769) and an interaction effect of negative self-views by panel member (*χ*^2^ (2) = 11.10, *p* = .004). Overall, participants liked the negative member less (*b* = -1.71, *SE* = 0.57, *t* = -3.01, *95% CI* [-2.83, -0.60], *std*.*b* = -0.46) and the positive member more (*b* = 1.62, *SE* = 0.57, *t* = 2.84, *95% CI* [0.50, 2.74,], *std*.*b* = 0.43) compared to the intermediate member. However, the interaction effect indicated that the tendency to like a panel member was dependent on level of negative self-views. Participants with more negative self-views reported lower liking of the positive member compared to the intermediate member (*b* = -0.35, *SE* = 0.17, *t* = -2.07, *95% CI* [-0.68, -0.02], *std*.*b* = -0.32), see Fig 4. Negative self-views did not affect the liking of the negative member compared to the intermediate member (*b* = 0.21, *SE* = 0.17, *t* = 1.25, *95% CI* [-0.12, 0.54], *std*.*b* = 0.20). In other words, participants liked the positive member more. However, participants with more negative self-views liked the positive member less, in line with our hypotheses. The variance explained in the model predicting liking by panel member, negative self-views and their interaction was *R*^*2*^ = 0.14, indicating a medium effect size, *Cohen’s f*^*2*^ = 0.17.

## Discussion

The current study sought to investigate whether negative self-views interfere with relating to others in the context of receiving character trait feedback. Specifically, this study aimed to explore whether negative self-views relate to how individuals affiliate with others that provide positive or negative character trait feedback on three outcome measures; closeness, trust and liking. First, findings from the panel version of the Social Feedback Task were in line with previous findings regarding mood, i.e., individuals feel better after positive feedback and worse after negative feedback, particularly when the feedback is not in line with their self-views [48]. In addition, with this version of the task we were able to show that overall, participants affiliated more with the member providing predominantly positive feedback and less with the member providing predominantly negative feedback, compared to the intermediate member.

Importantly, in line with our hypotheses, individuals with more negative self-views felt less affiliated with the panel member providing predominantly positive feedback on measures of closeness and liking. Moreover, individuals with more negative self-views reported lower mood in response to positive feedback. These findings may suggest that when people receive feedback that is not in line with their self-views, they may have difficulty affiliating with this person [18].

In the current study, it is likely that individuals with more negative self-views perceived the relevancy and applicability of positive feedback to their self-concept less well given they reported lower mood after positive feedback. They may therefore have been less able to form a sense of closeness and liking towards the positive member [16]. It should be noted that this pattern of difficulty in affiliating with positive others is observed in the context of interacting with a new person. This observation may indicate how pervasive these patterns of relating to the self and others are [10]. Consequently, new interactions are likely to reinforce negative self-views when it is challenging to learn from positive feedback and relate to positive others. An individual may become stuck in the inability to rely on the self (due to pervasive negative self-views) as well as the inability to rely on others e.g., epistemic freezing or fearful attachment [11, 53].

We sought to investigate negative self-views as an important aspect of identity disturbance in Borderline Personality Disorder (BPD) [20–22]. Our finding that individuals with more negative self-views have difficulty engaging with positive feedback and the positive member, may explain why people with BPD are less open to positive feedback [20, 71]. Indeed, the current finding supports research showing that individuals with BPD have difficulty engaging with positive social information and may explain why individuals with BPD may attend less to others after positive feedback compared to non-clinical controls in neuroimaging studies [20, 30, 33]. These challenges associated with negative self-views may not only be preventing individuals with BPD from learning positive aspects about themselves, but may also make it difficult for them to build relationships with people that view them in a positive light [23, 72]. As a result, negative self-views in BPD may encourage individuals to seek out less positive or supportive relationships [14, 31].

It is understood, however, that negative self-views are relevant to various psychological disorders [73] and these findings may be relevant when working with negative self-views in a clinical setting. It is possible that clinicians may be more inclined to support people with negative self-views by providing overly positive feedback or by getting patients to make positive self-statements. However, this study and other studies show that when positive feedback is not in line with the self-view, it may inadvertently lower mood and self-esteem [15]. Moreover, this study suggests that incongruent positive feedback could adversely affect the therapeutic alliance. The clinician may consequently experience this as the patient being distant and may find it more challenging to relate to the patient [37, 74, 75]. Instead, it may be helpful for clinicians to be curious about the person’s experiences with negative self-views and to validate the difficulties they may have in feeling understood and supported. Through understanding the person’s experiences, clinicians may foster the therapeutic relationship and a more compassionate view of self in patients.

While there was an effect of negative self-views on the level of closeness and liking towards the positive member, the effect was less apparent for the level of trust individuals displayed towards the positive member. Within the analyses, it was found that those with more negative self-views had less trust in the positive member, however, the finding did not survive multiple statistical correction. A potential explanation for the non-significant finding could be that a stronger deviation in trust building may be present in clinical samples such as people with a diagnosis of BPD (e.g. [36]). Participants may have also found it more difficult to endorse a sense of trust in the positive member compared to endorsing closeness and liking given the circumstances of the pandemic did not allow for participants to have a face-to-face real-life interaction with the panel members as part of the Social Feedback Task. Hagerty, Lynch-Sauer [54] suggest that in order to develop a sense of interpersonal affiliation with others it may require ‘active involvement’ with the other person. Future research could aim to employ a real-life social interaction in the Social Feedback Task with other participants or confederates as ‘panel members’ or study trust responses in clinical samples.

While negative self-views were associated with how individuals affiliated with the positive member in the Social Feedback Task, they did not appear to relate to how individuals affiliated with the negative member on the measures of closeness, trust and liking. In terms of affiliating with the negative member, the current findings suggest that there may be multiple processes at play. It could be the case that individuals with negative self-views affiliated more with someone who confirms their negative self-views [16, 18]. However, this effect may have been dampened by other processes relevant for the negative member that we did not observe in affiliating with the positive member. In the context of BPD, previous research has found that regardless of its consistency with their self-view, negative social feedback may still have a detrimental effect on individuals in terms of mood and relationships [20, 76, 77]. In other words, the confirmation of self-views may facilitate affiliation but only to the degree that negative feedback does not undo this effect. Alternatively, it could be thought that individuals with more negative self-views have different learning rates, i.e., they may have had more difficulty in learning the panel’s usual responding style. However, it was not the case that individuals with more negative self-views felt significantly less affiliated overall (i.e., there was no main effect of negative self-views on affiliation). This indicates that individuals with negative self-views do differentiate how they affiliate with the member providing predominantly positive compared to predominantly negative feedback. Previous studies have indicated that the ability to learn from others may depend on the degree to which people have internalised previous positive interactions with others [78]. Future research could investigate whether people with negative self-views are less volatile in their learning style. This would enrich the understanding around pervasiveness of negative self-views, i.e., whether people may be less likely to change their views on self and others.

A main strength of the current study was that using an experimental paradigm, we were able to show how negative self-views may interfere with building new relationships. The panel version of the validated Social Feedback Task resulted in the expected mood responses for negative and positive feedback compared to intermediate feedback as well as for feedback that was more congruent with the self-views. It was also effective in inducing different responses surrounding measures of closeness and liking towards the panel members, in particular the positive member. The findings from this task allow us to estimate how individuals may affiliate with others differently depending on the feedback they provide.

However, the limitations of the study must also be acknowledged. Firstly, the study used a non-clinical sample with participants recruited through a university population which may limit the generalisability of these findings. Significant identity disturbances and BPD features have found to be commonly present in university samples [79, 80]. In the current study, there was a broad range of self-reported BPD features including clinically relevant BPD features. Moreover, we confirmed in the current sample that negative self-views were strongly related to self-reported BPD features, in line with previous findings that BPD is associated with severe identity disturbances [20, 22, 81]. Nonetheless, the level of impairment is often more severe in clinical samples. Therefore, future research using clinical samples, such as patients diagnosed with BPD and matched controls, should aim to replicate these findings to see how these individuals relate to the different panel members. Moreover, findings may not be limited to BPD but may also be relevant to other disorders such as mood disorders [82, 83]. Investigating different psychiatric disorders in future research, may contribute to understanding whether this finding is specific to BPD or a transdiagnostic feature of negative self-views. Second, the sample size was relatively small. Using multilevel analysis, we were able to include all trials per participant as data points. However, replicating these findings using a larger sample size would be advised. Due to the covid-19 pandemic, another limitation was that the study was conducted online. Previous literature have indicated that there is an effect of feedback by others in people of the general population, even when they know the feedback is computerised [84]. Although most participants still believed the cover story of the procedure, conducting the study in-person with a real-life social interaction between participants and “panel members” may have made the feedback more meaningful and more likely to influence individuals’ level of affiliation.

In conclusion, the current study showed that the way in which individuals view themselves is associated with how they relate to others. The study found that individuals with more negative self-views felt less close to and reported lower liking for the positive panel member. Negative self-views did not appear to influence how individuals affiliated with the negative panel member. These findings suggest that when individuals with high negative self-views, such as those with BPD, are viewed by others in a positive light, they may not see themselves reflected and may find it difficult to affiliate with this person. Negative self-views may therefore adversely impact the building of interpersonal relationships. In a clinical setting, this may interfere with building a positive therapeutic relationship and may affect what the individual learns from therapy. An individual with negative self-views may find it difficult to take on positive feedback and form a close relationship to the clinician [85]. In clinical practice, it is recommended that clinicians have awareness of how negative self-views in the patient as well as their own reactions to the patient may affect the therapeutic relationship. Supportive and validation techniques may be helpful for the patient to feel understood and to build a therapeutic alliance but reactions to these techniques may need careful monitoring. A supportive, validating therapeutic alliance may in turn increase the patient’s acceptance of positive social interactions and improve their relationships outside of the therapy room.

## Supporting information

S1 File (PDF)
