# Supplementary material for: How negative self-views may interfere with building positive relationships: An experimental analogue of identity dysfunction in borderline personality disorder
Source: PLoS One. 2024 Mar 28;19(3):e0301196. doi: 10.1371/journal.pone.0301196 (PMC10977689; doi:10.1371/journal.pone.0301196)
Supplement: S1 File — (PDF) [file pone.0301196.s001.pdf]

Supplementary information

To manuscript titled "How negative self-views may interfere with building positive relationships: an experimental analogue of identity dysfunction in borderline personality disorder"

Interview Questions for SF Task

I will now hold a short interview with you. The interview recording will be shared with the panel members so that they can listen to learn more about you and will use this and your answers to make their impressions of you. There are no right answers so please try to answer as honestly as you can.

1. *What do you do in daily life?*
2. *What are your hobbies, interests?*
3. *How would your friends describe you? Which characteristics?*
4. *How would you describe yourself? Could you name 3 positive and 3 negative characteristics.*
5. *What are you proud of? What are you ashamed of?*
6. *If you could change one thing of yourself what would this be?*
7. *If you could be someone else for one day, who would you be and why?*
8. *What would you do in the following situation: You have just paid for your groceries and upon leaving the store you find the cashier returned two dollars too many.*
9. *What would you do in the following situation: There is a child crying in the street and no one is helping her. You have to take a bus which is just arriving, to visit a friend in hospital who will have surgery.*
10. *What would you do in the following situation: Your neighbour has Alzheimer and acts very anti-social. She is annoyed when you offer help and proud of her independence. However, she sometimes forgets to turn the stove of or to close the front door. As you walk out you see her door is open.*

S1 Table
Character Trait Words used in SF Task

**Negative**	**Intermediate**	**Positive**
Nasty	Chaotic	Friendly
Selfish	Critical	Confident
Arrogant	Slow	Healthy
Mean	Clumsy	Funny
Unreliable	Strict	Smart
Stupid	Nervous	Respectful
Lazy	Anxious	Kind
Cold	Weird	Grateful
Cowardly	Reserved	Honest
Harsh	Competitive	Courageous
Bossy	Serious	Warm
Pesky	Quiet	Generous
Boring	Talkative	Loyal
Stubborn	Neat	Sincere
Rigid	Agreeable	Caring

Manipulation Check Interview Questions for SF Task

1. *How did you experience this study?*
2. *What do you think of the panel members that provided you feedback?*
3. *If you could meet one of the panel members, who would you want to meet and why?*
4. *Has the feedback affected you in any way?*
5. *To what extent do you think the interview sufficed to get an impression of you?*
6. *How confident are you that the feedback was provided by these panel members?*

S2 Table
Distribution of positive, intermediate and negative feedback among the three panel members

Feedback valence	Panel member 1 – predominantly negative	Panel member 2 – intermediate	Panel member 3 – predominantly positive	Subtotal
Positive	2	3	10	15
Intermediate	3	9	3	15
Negative	10	3	2	15
Subtotal	15	15	15	45

S3 Table
Counterbalance orders

Version	V1	V2	V3	V4	V5	V6
Negative member	1	2	3	1	3	2
Intermediate member	2	3	1	3	2	1
Positive member	3	1	2	2	1	3

Note: names of the panel members were matched to the sex of the participant. Female names were: 1) Rose, 2) Sarah, 3) Mia. Male names were: 1) Christian, 2) Tyson, 3) Ethan.

S4 Table
Parameters of model predicting mood by valence, applicability, panel member and valence by applicability interaction

	Estimate	Std. Error	t value	95%CI lower bound	95%CI upper bound	Std.b
Intercept (Intermediate valence)	4.69	0.21	22.21	4.28	5.09	0.00
Negative valence	**-1.19**	**0.16**	**-7.30**	**-1.50**	**-0.88**	**-0.28**
Positive valence	**0.80**	**0.23**	**3.43**	**0.35**	**1.25**	**0.19**
Applicability	**0.26**	**0.03**	**10.25**	**0.21**	**0.31**	**0.27**
Negative member	**-0.16**	**0.08**	**-2.01**	**-0.32**	**-0.01**	**-0.04**
Positive member	0.01	0.08	0.09	-0.15	0.16	0.00
Negative valence*Applicability	**0.12**	**0.04**	**3.19**	**0.05**	**0.19**	**0.11**
Positive valence*Applicability	0.00	0.04	-0.01	-0.08	0.08	0.00

S5 Table
Parameters of model predicting mood by valence, applicability, panel member, negative self-views and two-way interactions of valence by applicability, negative self-views by applicability and negative self-views by valence

	Estimate	Std. Error	t value	95%CI lower bound	upper bound	Std.b
Intercept (Intermediate valence)	3.95	0.73	5.45	2.53	5.36	0.00
Negative valence	**-1.25**	**0.31**	**-3.98**	**-1.87**	**-0.65**	**-0.29**
Positive valence	**1.77**	**0.39**	**4.55**	**1.02**	**2.56**	**0.41**
Applicability	**0.57**	**0.06**	**8.99**	**0.44**	**0.69**	**0.60**
Negative self-views	0.26	0.22	1.21	-0.17	0.68	0.11
Negative member	-0.35	0.29	-1.18	-0.92	0.23	-0.08
Positive member	-0.22	0.29	-0.74	-0.78	0.35	-0.05
Negative valence*Applicability	0.07	0.04	1.81	0.00	0.15	0.06
Positive valence*Applicability	-0.01	0.04	-0.36	-0.09	0.06	-0.02
Negative valence*Neg self-views	0.07	0.10	0.70	-0.12	0.26	0.05
Positive valence*Neg self-views	**-0.28**	**0.09**	**-3.04**	**-0.47**	**-0.10**	**-0.23**
Negative member*Neg self-views	0.06	0.09	0.66	-0.11	0.23	0.05
Positive member*Neg self-views	0.07	0.09	0.75	-0.10	0.23	0.05
Applicability*Neg self-views	**-0.10**	**0.02**	**-5.45**	**-0.14**	**-0.06**	**-0.42**

S6 Table
Parameters of model predicting connectedness by panel member and negative self-views

	Estimate	Std.Error	t value	95% CI lower bound	95% CI upper bound	std.b
**Closeness**						
Intercept (Intermediate member)	3.51	0.89	3.95	1.76	5.30	0.00
Negative member	**-1.37**	**0.52**	**-2.63**	**-2.41**	**-0.34**	**-0.33**
Positive member	**1.46**	**0.52**	**2.79**	**0.42**	**2.49**	**0.35**
Negative self-views	0.14	0.26	0.55	-0.37	0.68	0.07
Negative member*Neg self-views	0.18	0.15	1.19	-0.12	0.49	0.15
Positive member*Neg self-views	**-0.31**	**0.15**	**-2.01**	**-0.61**	**-0.01**	**-0.26**
**Trust**						
Intercept (Intermediate member)	4.73	0.83	5.67	3.06	6.34	0.00
Negative member	-1.14	0.57	-2.01	-2.27	0.01	-0.28
Positive member	**1.61**	**0.57**	**2.83**	**0.44**	**2.72**	**0.39**
Negative self-views	-0.06	0.25	-0.26	-0.54	0.42	-0.03
Negative member*Neg self-views	0.13	0.17	0.75	-0.21	0.47	0.10
Positive member*Neg self-views	-0.33	0.17	-1.99	-0.66	0.01	-0.28
**Liking**						
Intercept (Intermediate member)	5.58	0.64	8.68	4.31	6.82	0.00
Negative member	**-1.71**	**0.57**	**-3.01**	**-2.83**	**-0.60**	**-0.46**
Positive member	**1.62**	**0.57**	**2.84**	**0.50**	**2.74**	**0.43**
Negative self-views	-0.003	0.19	-0.01	-0.37	0.37	0.00
Negative member*Neg self-views	0.21	0.17	1.25	-0.12	0.54	0.20
Positive member*Neg self-views	**-0.35**	**0.17**	**-2.07**	**-0.68**	**-0.02**	**-0.32**
